# A colonial serrated polyp classification model using white-light ordinary endoscopy images with an artificial intelligence model and TensorFlow chart

**DOI:** 10.1186/s12876-024-03181-3

**Published:** 2024-03-05

**Authors:** Tsung-Hsing Chen, Yu-Tzu Wang, Chi-Huan Wu, Chang-Fu Kuo, Hao-Tsai Cheng, Shu-Wei Huang, Chieh Lee

**Affiliations:** 1 Department of Gastroenterology and Hepatology, Linkou Medical Center, Chang Gung Memorial Hospital, Taoyuan, Taiwan; 2 College of Medicine, Chang Gung University, Taoyuan, Taiwan; 3 Business Futures Co., LTD, Tokyo, Japan; 4 Division of Rheumatology, Allergy, and Immunology, Chang Gung Memorial Hospital- Linkou and Chang Gung University College of Medicine, Taoyuan, Taiwan, ROC; 5 Center for Artificial Intelligence in Medicine, Chang Gung Memorial Hospital, Taoyuan, Taiwan, ROC; 6 Division of Gastroenterology and Hepatology, Department of Internal Medicine, New Taipei Municipal TuCheng Hospital, New Taipei City, Taiwan; 7 Graduate Institute of Clinical Medicine, College of Medicine, Chang Gung University, Taoyuan City, Taiwan; 8 Department of Information and Management, College of Business, National Sun Yat-sen University, Kaohsiung city, Taiwan

**Keywords:** Colonial polyps, Serrated-type colon polyps, Artificial intelligence, Classification modeling

## Abstract

In this study, we implemented a combination of data augmentation and artificial intelligence (AI) model—Convolutional Neural Network (CNN)—to help physicians classify colonic polyps into traditional adenoma (TA), sessile serrated adenoma (SSA), and hyperplastic polyp (HP). We collected ordinary endoscopy images under both white and NBI lights. Under white light, we collected 257 images of HP, 423 images of SSA, and 60 images of TA. Under NBI light, were collected 238 images of HP, 284 images of SSA, and 71 images of TA. We implemented the CNN-based artificial intelligence model, Inception V4, to build a classification model for the types of colon polyps. Our final AI classification model with data augmentation process is constructed only with white light images. Our classification prediction accuracy of colon polyp type is 94%, and the discriminability of the model (area under the curve) was 98%. Thus, we can conclude that our model can help physicians distinguish between TA, SSA, and HPs and correctly identify precancerous lesions such as TA and SSA.

## Introduction

**Fig. 1:**
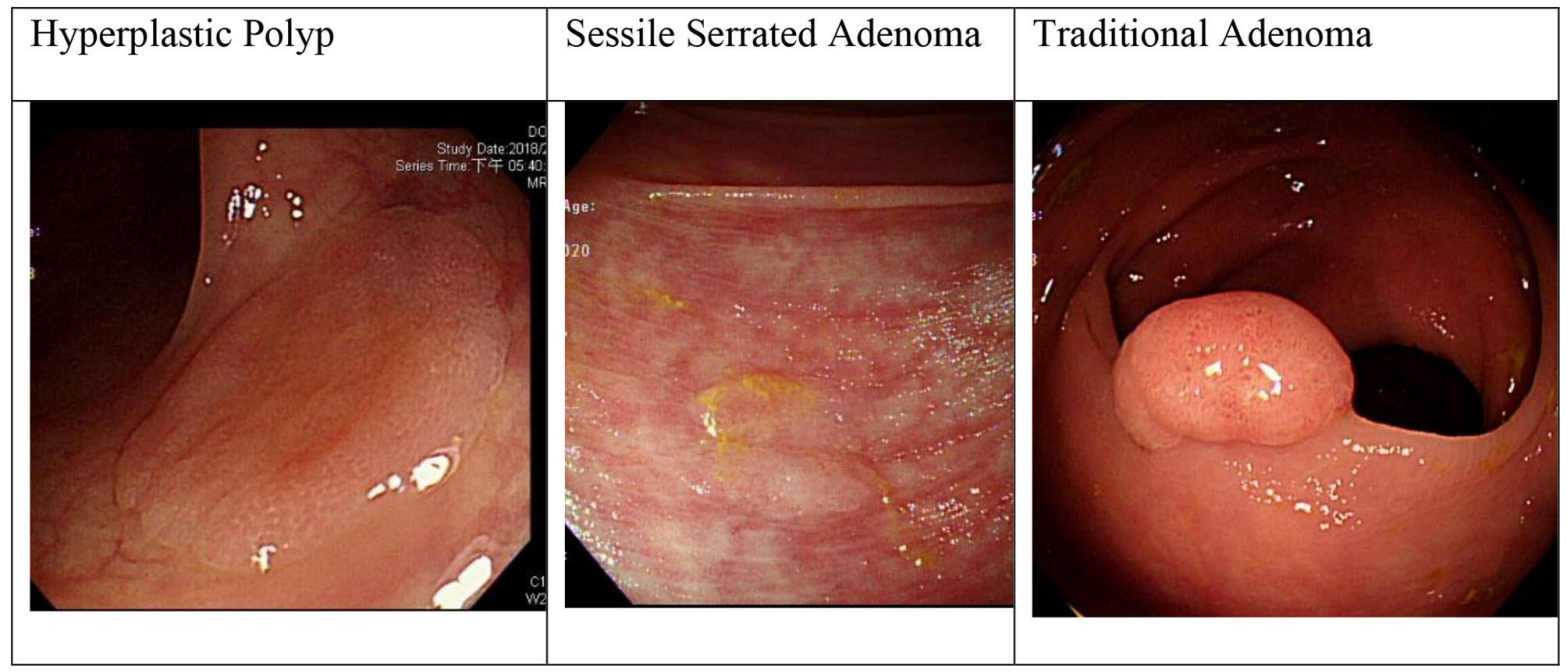
HP, SSA, and TA under white Light

Colon cancer is the leading cause of death globally. Researchers discovered evidence that removing precancerous lesions can efficiently reduce the development of colon cancer and the medical burden associated with it [1]. Colonoscopy can assist physicians in visually identifying the lesions in the colon, but it relies on physicians to determine whether it is a precancerous lesion. This study focuses on classifying sessile serrated adenomas (SSA), traditional (conventional) adenomas (TA), and hyperplastic polyps (HP), as 90% of colorectal cancers arise from colon adenomas. The serrated type of colon polyps includes traditional serrated adenoma (TSA), sessile serrated adenoma (SSA), and hyperplastic polyp (HP). TSA and SSA are precancerous lesions that account for 15–30% of colorectal cancers [2, 3]. As shown in Fig. 1, compared with the non-harmful lesion, hyperplastic polyps (HP), the TSA has a prominent appearance such as reddish, protruded, pedunculated, “pinecone-like” or “branch coral-like” lesions in white light endoscopic view. Hence, it is easy to distinguish TSA from HP, even with traditional adenoma (TA). Current studies, such as those by Brown et al. [4], have developed efficient deep-learning models to help endoscopists locate the TA and SSA. However, in their study, they did not classify the observed polyps including TA, SSA and HP in a single framework.

In contrast to TSA, SSA does not have such precise characteristics to distinguish itself from HPs. While endoscopists can remove all observed polyps, removing polyps with endoscopic treatment involves risks and a financial burden. Internal bleeding, perforation, and subsequent bleeding are all common risks associated with the polyp removing procedure. The financial costs involved in lesion removal are also substantial. Hence, patients and the healthcare industry would like to improve the rate of correctly classifying the type of polyp and remove the SSA, TSA, and TA but not the HP.

The current practice of increasing the classification rate of lesion types is to train endoscopists with past endoscopy images with and without narrowband images (NBI). However, such a training process takes several years, and its duration can vary significantly from one physician to another. For example, Lee et al. [5] reported a new one-year comprehensive training program for an endoscopist workgroup to increase their ability to classify serrated polyps from other lesions correctly. The healthcare industry needs an efficient and consistent method to help physicians classify the lesion type as they locate the lesion via endoscopy. The artificial intelligence (AI) of image pattern recognition shows promising potential to achieve this goal. Artificial intelligence has been applied to classify colon neoplasm to no neoplasm [6, 7].

However, the SSA classification remains under-studied. Furthermore, accurately classifying the SSA, TA, and HP more than often requires enhanced images such as NBI and/or magnified endoscopy. For example, Azam et al [8] built an AI model with both wight-light and NBI images to improve the accuracy of their model. However, the NBI or other enhanced method has their weaknesses [9]. The efficiency of NBI is highly dependent on bowel preparation, and the image of NBI is darker than the white-light images. Other enhanced methods, such as magnified endoscopy might not be available. Therefore, using enhanced images to build an AI classification model can significantly limit the generality of the model. Nemoto et al [10] built an AI model that can distinguish SSA and TA using white-light images. Their accuracy ranges from 77 to 87%.

Among all the artificial intelligence methods, CNN is the most widely used for image classification. Hirasawa et al. [7] used a CNN to classify 2296 endoscopy images into gastric and non-gastric cancers and correctly identified 71 out of 77 cases. There are several different CNN-based classification models that can be implemented in the Tenser-Flow chart. The most popular CNN-based models include ResNet 50/ResNet 34, MoblieNetV2, GoogLeNet, AlexNet, etc., all of which have been applied to build a classification model [11]. Other than those well-known CNN-based models, there are several advanced models for colonoscopy polyp classification such as ResUNet++ (cf. Jha et al., [12]), TGANet (cf. Tomar et al [13]), DoubleUNet (cf. Lin et al. [14]), Polyp-PVT(cf. Dong et al. [15]), and PraNet (cf. Fan et al. [16]). However, those more advanced models aim to segment polyps with a wide range of features and hence require more training samples, thus not suitable for our study. In this study, we intend to propose an AI modeling framework that can be implemented in any generic healthcare institution. Therefore, we choose to implement a more stable and well-received CNN-based deep-learning model and combined with a data argumentation heuristic to reduce both of the technical barrier and input sample size.

Among those well-received CNN-based deep-learning models, by incorporating residual learning in the traditional neural network, the ResNet model is known for its high accuracy without overfitting [17]. However, the computational cost of ResNet is relatively high, which limits the application of ResNet model as one needs to embed multiple AI models in one system and/or instant result. In this study, the polyp classification model should be embedded in the endoscopy system, and an instant classification result is needed during the endoscopy section. Furthermore, Nemoto et al. [10] use ResNet50 as their modeling backbone in distinguish the SSA and HP and they find that their AI model with ResNet50 cannot outperform the trained physicians. Therefore, the ResNet model might not be the best fit for classifying the colonic polyps. Inception V4 is designed to reduce the computational cost with state-of-art accuracy [18]. As stated by McNeely-White et al. [19], the Inception and ResNet model’s performances are very similar, while the Inception requires less computation power. To further improve the speed of computation, MoblieNetV2 is proposed to build a light-weight model [20]. According to Canziani et al. [11]’s comprehensive review, Inception V4 has the highest accuracy with a moderate number of parameters. In conclusion, the Inception V4 is best fit of our study.

In this study, we propose an AI modeling framework that can help any healthcare institution to classified SSA, TA, and HP with a single model and use only white-light images. Our novelty of the AI modeling framework lies on two aspects. First, to overcome a problem with small sample size of SSA images, we construct a deep-learning model combined with an image-preprocessing algorithm. Second, to make the AI model more generally applicable across different endoscopies, our AI modeling framework requires only ordinary white-light images. The proposed model is a highly efficient model that classified TA, SSA, and HP with an average accuracy rate of 94.43%, a sensitivity of 98.62%, a specificity of 97.12%, and an AUC of 97.87%. Our model required a limited sample size and only non-magnified white-light images.

## Materials and methods

This study was approved by the Institutional Review Board of the Chang Gung Memorial Hospital (IRB No. 202001328B0). Polyps were detected by ordinary white-light endoscopy. We collected 257 images of HP, 423 images of SSA, and 60 images of TA under white light. We also collected 238 images of hyperplastic, 284 images of SSA, and 71 images of TA under NBI. It is worth noting that our data collection does not aim to reassemble a data set according to the actual proposition of SSA, TA, and HP. By contrast, our data collection method aims to allow the AI model to learn different characteristics of SSA, TA, and HP to classify them. During the data collection stage, we intentionally collected the data using an unequal sampling method. Unequal sampling is a commonly used statistical method for this purpose. Uneven sampling broadens the proposition of defects or target items and enables the statistical model to extract characteristics from the target group. In this study, we implemented unequal sampling to increase the proportion of SSA and enable the AI model to extract features from SSA images. Experienced endoscopists classified all images and pathologies and verified all classification results. TensorFlow (https://www.tensorflow.org/) with Inception V4 deep-learning model are implemented to construct our deep learning model. Images of colon polyps were collected from the Lin-Kou Chang Gung Memorial Hospital database between 2016 and 2019. Two experienced gastrointestinal pathologists reviewed the pathology of colon polyps. Images with blurred surfaces and poor focal lengths were discarded. The resolution of the images was 150 × 150 bpi. Our deep learning heuristic consisted of three parts: the data augmentation algorithm, deep learning framework, and CNN model. At the end of this section, we present our statistical validation methods and operational environment.

### Image Preprocessing

To address this small sample size, we developed a procedure to increase the sample size of the images. Data augmentation is an effective and commonly applied method for defect detection [21]. We adopted a similar idea to design our image preprocessing algorithm. The collected images first go through the preprocessing algorithm (*Algorithm 1*), and then the deep learning model is built, as described in *Heuristic 1*.

The image preprocessing algorithm.

Indices.

i = the *i*^th^ deep learning dataset.

*k* = the *k*^th^ randomly divided sub dataset in k-fold cross validation method.

#### Algorithm 1

Step 1: Process the images into the correct input format for the TensorFlow.

**Fig. 2:**
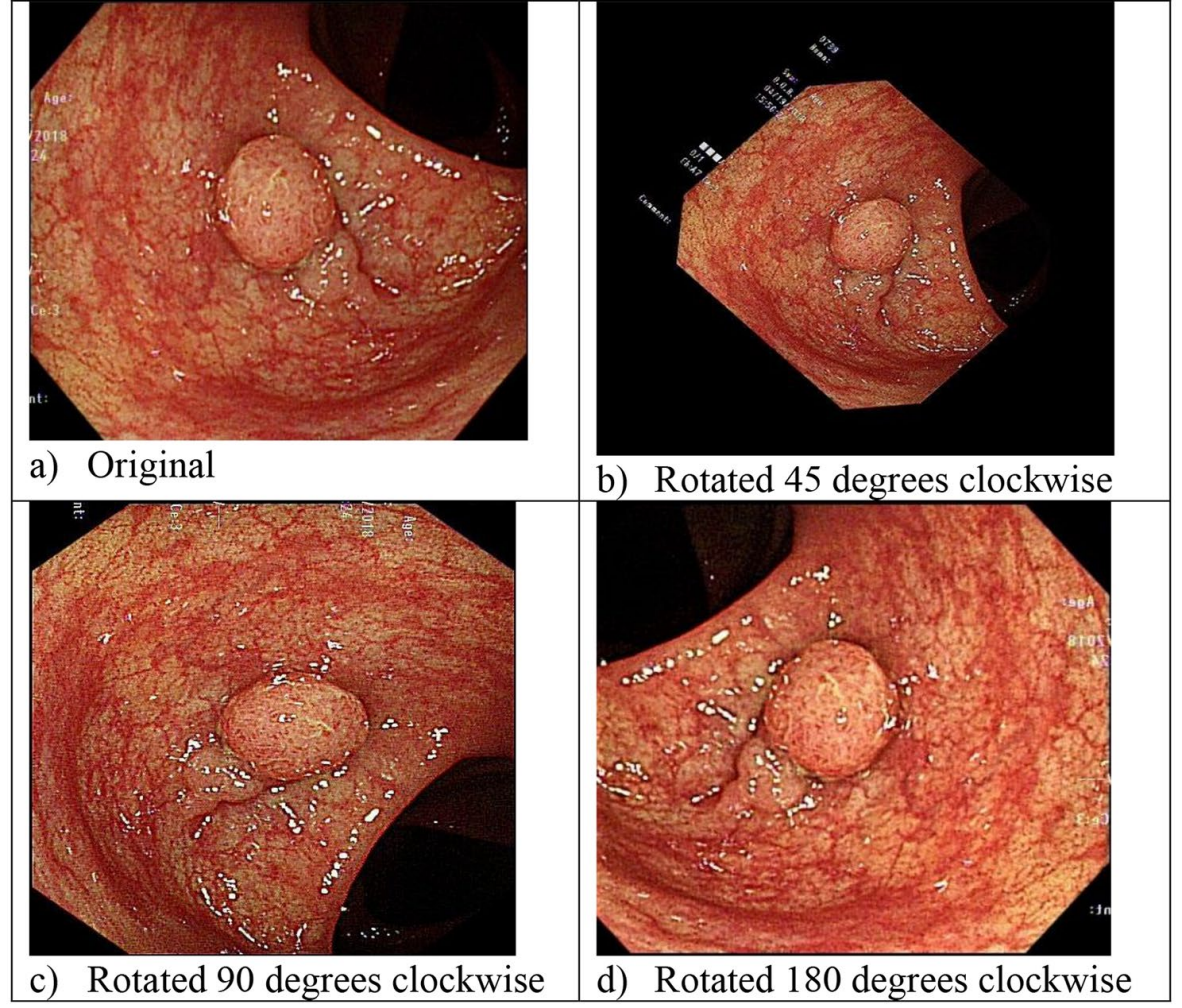
Rotated and enhanced images

Step 2: Augment the images with rotation. For example, one can rotate images by 45°, 90°, and 180°, and triple the new useable images. The images are also enhanced by image enhancement software (see Fig. 2).

Step 3: Collect and randomly divide the images into four equal-sized subsets within each type of polyp. Assigned each subset with an index number *k*. For example, in 4-fold cross validation there are four subsets, *k =* 1, 2, 3, and 4.

Step 4: Construct deep-learning datasets. Every dataset was constructed using the training, validation, and testing sets. All deep learning datasets were named with the subsets generated in Step 3. In 4-fold cross validation, there are 12 heterogeneous deep-learning datasets, that is *i* = 1.12.

Step 5: Output the deep learning building datasets into *Heuristic 1*. End the *Algorithm 1*.

Nate that, we named each deep leaning dataset *i* based on the subset index *k*. For example, if a training set consisted of subsets 1 and 2, a validation set is of subset 3, and a test set is subset 4. The deep learning dataset was named 1234.

### Framework of TensorFlow

In this study, TensorFlow was conducted in the Anaconda environment using Jupyter Notebook and Python. We used a CNN model, called Inception V4, which includes Softmax, Dropout, Average Pooling, Inceptions, and Reduction layer. The basic idea of inception includes multiple convolution layers, average pooling layers, and activation functions (such as Rectified Linear Unit (ResLU)). Softmax and dropout were used to prevent model overfitting. The convolution layer extracts characteristics from the image. The activation functions introduced weights for the standard deviations and added a small value to the bias [21]. The active functions can help generate a nonlinear combination of the convolution layer and thus activate the neurons and avoid dead neurons. The pooling layer retained significant characteristics and avoided overfitting problems [22] These three parameters—learning rate for the activation function, batch size, and epoch for convolution—must be optimized within the deep learning process. Detailed information on Inception V4 can be found in the study by Szegedy et al. [18]. We implemented Inception V4 as the convolution neuron network model because of its consistency and performance in our preliminary modeling experiments. The deep learning model-building procedure is summarized in *Heuristic 1*.

The Heuristic of the deep learning model building.

#### Heuristic 1

Step 1: Collect data and mark the true state of the images.

Step 2: Input data into *Algorithm 1*.

Step 3: Index the deep learning datasets from *i =* 1*…* 12 in 4-fold cross validation method. For convenience, we label subsets for each deep learning dataset as *j =* 1,2,3,4. Note that *j* represented the order of the subsets in the deep learning dataset, and it is not quals to *k*.

Step 4: initiate the Heuristic by set *i =* 1 and go to step 5.

Step 5: Input deep learning dataset *i.* Go to step 6.

Step 6: Input subsets *j =* 1, 2, and 3 into TensorFlow model. The subsets *j* = 1 and 2 are training sets, and the validation set is set *j =* 3. Find the best parameters (learning rate, batch size, and epoch) for the deep learning model in Step 6, and output the model to Step 7.

Step 7: Input subsets j = 4 into the deep learning model built in Step 7 to test its accuracy. Record the testing results for deep learning dataset *i*.

Step 8: Collect the model testing results. If *i = n*, stop and output all testing results from the deep learning models. Otherwise, set *i = i* + 1 and return to Step 5.

Step 9: Collected all the testing results for statistical analysis.

**Fig. 3:**
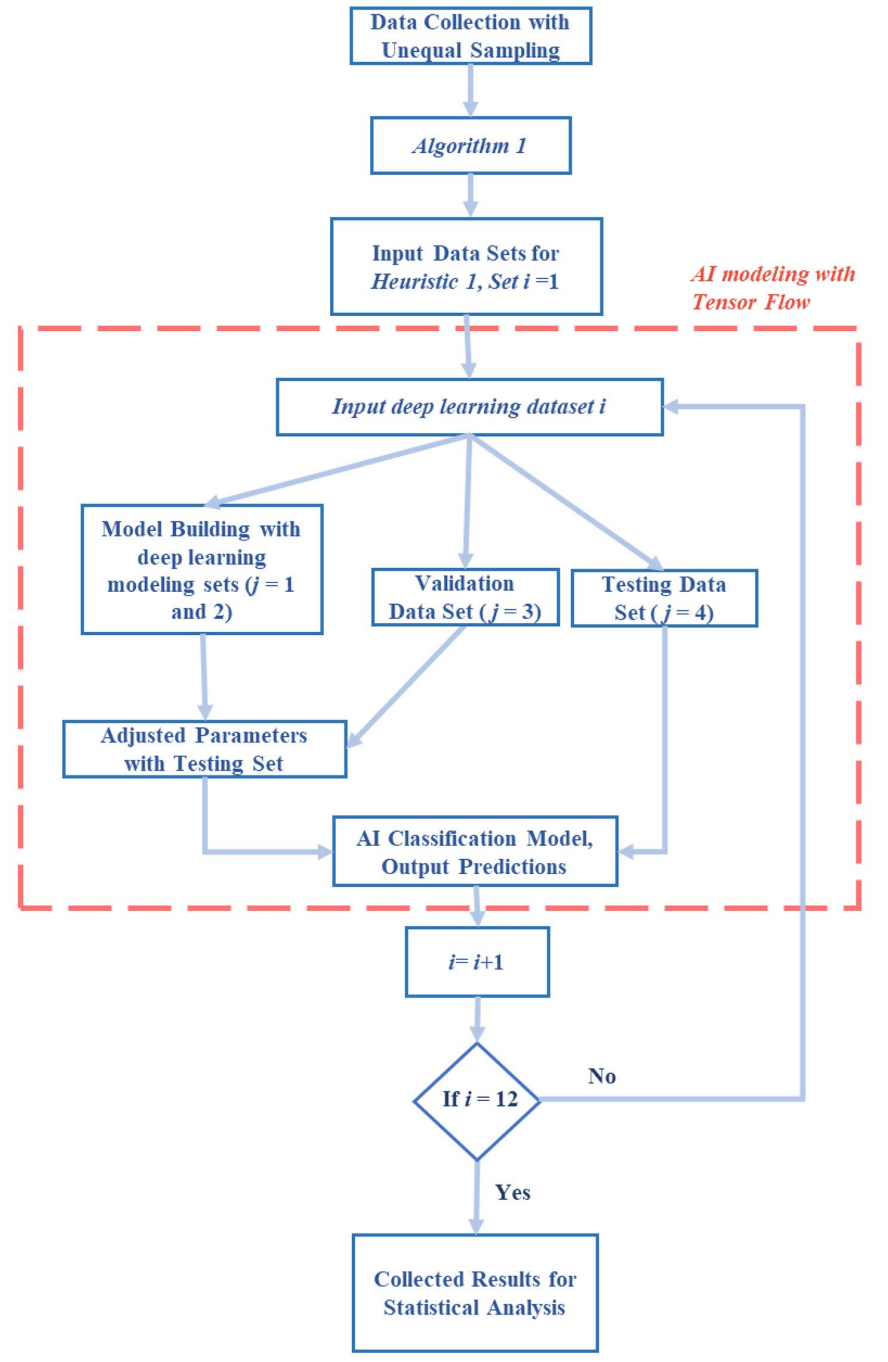
AI modeling framework

In Fig. 3 below, we present our overall AI modeling framework.

### Statistical analysis

A percent confidence interval analysis was implemented to benchmark the consistency of the deep learning model. To highlight the classification power of our deep learning model, discriminability indicators such as sensitivity, specificity, and area under the curve (AUC) were calculated. We also present a confusion matrix to summarize these indicators. All statistical analyses were conducted using Phyton 3.7.

A confusion matrix was constructed by defining the correct classification if the model could identify an image containing HP or adenoma. That is, if an SSA image is classified as TA by our model in our confusion matrix, it is still recorded as a true positive, and vice versa. In contrast, if an SSA(TA) image is classified as HP by our model, then we record it as a false negative, and vice versa (see Table 1).

**Table 1:** Confusion matrix

		True Condition	
		SSA/TA	HP
Predicted Condition	SSA/TA	True Positive	False Positive
	HP	False Negative	True Negative

From the calculation of the confusion matrix, we can calculate sensitivity and specificity as Eqs. (1) and (2), respectively. The result of the sensitivity and specificity and the area under the curve (AUC) serve as an indicator of the model discriminability.

1 $$Sensitivity = \frac{{True\,Positive}}{{True\,Positive + False\,Negative}}$$

2 $$Specificity = \frac{{True\,Negative}}{{True\,Negative + False\,Positive}}$$

As described in the method, we used the classical 4-fold method to check for overfitting in the deep learning model. A 4-fold method is a commonly adopted cross-validation method for deep learning. A 5-fold or even k-fold method can be used. We adopted the 4-fold method for its simplicity and efficiency. As described in *Heuristic 1*, the augmented data were randomly split into 4 equal-size subsets. Two of the subsets were assigned to build the model: one subset served as a validation set to set the parameters, and one test was set to test the accuracy of the final model. The results were recorded and assessed based on accuracy, sensitivity, specificity, and AUC. In addition to the 4-fold method, we also validated our AI model with two different aspects including other popular method such as ResNet50 and MobileNetV2 instead of Inception V4, and enhanced coloscopy images such as NBI images.

Since we aimed to design a method for healthcare institutes to build their own deep learning model, our method should be easy to build and execute. The Inception V4 is a stable and cost efficient deep-learning model and the white-light images are available for any brand of endoscopy. The execution environment is summarized in Table 2. As can be observed, our execution environment requirements are affordable for any healthcare institution. This is another advantage of the proposed method.

**Table 2:** The hardware and software environment of deep learning model

	Item	Content
Hardware	GPU	Tesla P100
	GPU RAM	16.0 GB
Software	Windows	Windows 10 pro
	Operation System type	x64 processer
	NVIDIA	NVIDIA 441.22
	CUDA	CUDA 10.2
	Language Environment	Anaconda3 Jupyter Notebook
	Language	Python 3.7
	TensorFlow version	TensorFlow-GPU 1.14

## Results

### Results for the proposed AI modeling framework

The input images of TensorFlow are the outputs of the preprocessing algorithm (*Algorithm I*), and in this section, we only present the results for white-light images. The initial deep-learning building datasets are summarized in Table 3, and the execution environment is summarized in Table 2.

**Table 3:** The total number of input images for different subsets

		Hyper	SSA	Ademola
White light	Training	514	846	118
	Validation	257	423	59
	Testing	257	423	59
NBI	Training	506	568	142
	Validation	238	284	71
	Testing	238	284	71

The parameter selection was based on the results of the validation set. We discovered that the optimal validation accuracy occurred when the learning rate was 0.0001, batch size was four, and epoch was 77. The validation accuracy was approximately 95%. The learning rate and batch size were applied to all the deep-learning building datasets. Each set automatically optimized the number of epochs. Potential parameters are shown in yellow. We selected a batch size of 4, a learning rate of 0.0001, and epoch of 77 as our optimal model parameters based on validation accuracy. It is worth noting that the accuracy of the AI model for NBI did not outperform that of white light images. Because white exhibits promising accuracy, we decided to pursue an AI model that makes a classification with only white-light images. This result contradicts with that found in the current literature. Azam et al. [8] used an AI model to detect laryngeal squamous cell carcinoma; the white light and NBI images both showed promising accuracy. We hypothesize that this difference is because of image augmentation and pre-processing (*Algorithm 1*). White light images may contain more features and can be enhanced by image processing. While it is possible to process the images further, NBI’s AI model classification performance might improve. However, in practice, the AI model with white-light images as input is better than that with NBI. NBI requires switching lights and suffers from a focusing problem. In this research, we focus only on white-light images. Table 4 The parameter selection and the validation results. Note that while the training time is report, the testing time per image is almost instant after the model is built since the image is classified one-by-one by the resulting AI model.

**Table 4:** Parameters tested by validation process

Learning rate	Batch size	Epoch	Validation Accuracy	Training Time
0.045	4	6	0.57472825	160 to 170 s
0.01	4	6	0.57472825	160 to 170 s
0.005	4	6	0.57472825	160 to 170 s
0.001	4	6	0.57472825	160 to 170 s
0.0005	4	6	0.57472825	160 to 170 s
0.0001	4	77	0.95788044	160 to 170 s
0.0001	8	79	0.94972825	130 to 140 s

As shown in Table 5, for all deep learning models, the accuracy of the validation sets ranged from 94.15 to 96.33%, with an average accuracy of 95.34%. This shows that deep learning models hold consistently high accuracy for the validation sets. The model-building method was consistently effective. We also observed that the number of epochs varied from dataset to dataset, but the accuracy remained consistent. In other words, our deep learning heuristic helps us produce stable results across different deep learning datasets. A 99% confidence interval (CI) is added to illustrate the stability of our results. As shown in Table 5, the 99% CI ranges from 94.78 to 95.91%. The values of the datasets 1324 and 1423, 1432, and 2413 were below the confidence interval. However, even the lowest accuracy rate of 94.16% indicates that the model’s accuracy is promising.

**Table 5:** The accuracy rate for the validation sets of each dataset

Deep learning dataset name	epoch	Validation accuracy rate
1234	69	0.96195650
1243	66	0.95788044
1324	66	0.94429350
1342	69	0.95380437
1423	93	0.94701087
1432	86	0.94565219
2314	83	0.96331519
2341	95	0.96059781
2413	97	0.94157606
2431	97	0.95788044
3412	85	0.95788044
3421	39	0.94972825
	Average	0.95346467
	Std.	0.007512
	99% CI	0.9534 ± 0.005586

Table 6 summarizes the accuracy of the testing sets and the number of misclassifications for each testing set. The testing accuracy ranged from 93 to 96%, with an average of 94.43%. The 99% confidence interval ranged from 93.73 to 95.12%. Datasets 1432, 2413, and 3412 were below the confidence interval. Note that among these models, two out of the three models use subset 2 as the testing set. Hence, subset 2 may contain images that are difficult to classify correctly.

**Table 6:** The accuracy of testing sets

Deep learning dataset name	Testing accuracy	Misclassification
1234	0.9472	39
1243	0.9459	40
1324	0.9405	44
1342	0.9459	40
1423	0.9445	41
1432	0.9323	50
2314	0.9513	36
2341	0.9608	29
2413	0.9310	51
2431	0.9472	39
3412	0.9310	51
3421	0.9540	34
Average	0.9443	41.1667
Standard Deviation	0.0093	6.8601
99% CI	0.9443 ± 0.0069	41.17 ± 4.3587

Since we classified the lesions into three classes, the model might have misclassified SSA as adenoma. The misclassification types and their percentages among all misclassifications are summarized in Table 7. The percentage misclassification was calculated as the number of images of the misclassification type in the current testing set over the total number of misclassifications in the current testing set. As shown in Table 6, the most unwanted misclassification of SSA or adenoma as HP only consists of a small part (16% on average) of all misclassification types. While the classification power of our model to separate SSA and TA is superior to only classifying adenoma and HP, we should investigate the discrimination ability of TA and HP.

**Table 7:** The percent of each type of misclassification among all types of misclassification

Deep learning dataset name	SSA and TA were misclassified as HP	HP misclassified as SSA or TA	SSA and TA self-misclassified
1234	2.56%	17.95%	79.49%
1243	15.00%	25.00%	60.00%
1324	13.64%	11.36%	75.00%
1342	22.50%	25.00%	52.50%
1423	17.07%	17.07%	65.85%
1432	22.00%	18.00%	60.00%
2314	11.11%	19.44%	69.44%
2341	24.14%	13.79%	62.07%
2413	15.69%	33.33%	50.98%
2431	15.38%	5.13%	79.49%
3412	21.57%	15.69%	62.75%
3421	11.76%	8.82%	79.41%
Average	16.04%	17.55%	66.42%
Standard Deviation	6.08	7.69	10.18
95% CI	± 3.86	± 4.89	± 6.47

As Table 8 shows, the sensitivity ranged from 0.9772 to 0.9979, with an average of 0.9862. The standard deviation of the sensitivity was small, which indicates that our results are consistent. The specificity of our model ranged from 0.9339 to 0.9883, with an average value of 0.9712. The AUC was also high, with an average value of 0.9787. All three discrimination indicators demonstrate that our model performs consistently and accurately. Notably, the sensitivity of datasets 1432 and 3412 was lower than the 95% CI, yet still over 97%.

**Table 8:** Sensitivity and Specificity for each dataset

Deep learning dataset name	Sensitivity	Specificity	AUC
1234	0.9979	0.9728	0.9853
1243	0.9876	0.9611	0.9743
1324	0.9876	0.9805	0.9841
1342	0.9813	0.9611	0.9712
1423	0.9855	0.9728	0.9791
1432	0.9772	0.9650	0.9711
2314	0.9917	0.9728	0.9822
2341	0.9855	0.9844	0.9850
2413	0.9834	0.9339	0.9586
2431	0.9876	0.9922	0.9899
3412	0.9772	0.9689	0.9730
3421	0.9917	0.9883	0.9900
Average	0.9862	0.9712	0.9787
Standard Deviation	0.0060	0.0155	0.0093
95% CI	± 0.0038	± 0.0098	± 0.0059

### Results for variants of the proposed AI modeling framework

To enrich our validation analysis, we compare several variants of the proposed AI framework. We first conduct the analysis under same AI modeling framework while inputting the NBI images instead of wight-light images. Second, we implement other popular CNN-based AI models such as ResNet 50 and MobileNetV2 to further illustrate the suitability of the Inception V4.

Traditionally, endoscopists have used NBI to help classify polyp types. Thus, we validate our AI modeling framework with NBI images. The parameter selection and validation results are listed in Table 9. As we can observed from Table 9, our white-light image model outperformed the NBI images. As discussed in the NBI is not available in every hospital or clinic and exhibits low accuracy. We conclude that our white-light model is sufficient for building a polyp classification model. Thus, in this study, we found that without NBI images, we could still build a deep-learning model with high discriminability.

**Table 9:** NBI images model building accuracy results

learning rate	batch size	Epoch	Validation Accuracy rate	Training time
0.0001	4	83	0.88682431	About 13 to 14 s.
0.00005	4	94	0.87500000	About 13 to 14 s.
0.0001	4	119	0.88513511	About 13 to 14 s.
0.00005	4	147	0.88513511	About 13 to 14 s.
0.0001	8	47	0.88851351	About 13 to 14 s.
0.00005	8	142	0.88851351	About 13 to 14 s.

There is various other CNN-based classification models that can be implemented in the Tenser-Flow. As aforementioned, the ResNet50 is known to aim for higher accuracy while requiring more computation power, and the MobileNetV2 aims for acceptable accuracy while requiring limited computation power. We select Inception V4 for its stability, high accuracy, and limited size of parameters. To further validate the suitability of Inception V4, we conduct a comparison study between the Inception V4, ResNet50, and MobileNetV2. Table 10 summarizes the performance of the parameter selection validation data set. From Tables 9 and 10, we conclude that our proposed AI model framework is at least on par with other popular CNN-models base model and/or with enhanced images.

**Table 10:** Accuracy of other CNN-based models

Model	Data Set	Accuracy
Inception V4	Validation	0.9497
ResNet 50	Validation	0.9267
MobileNetV2	Validation	0.9233

## Discussion

The adenoma–carcinoma sequence was first described by Morson [23] in 1974. The removal of all colonic precancerous lesions during colonoscopy is a consensus worldwide. The “resect and discard” policy is recommended not only to reduce the risks of colon polyp removal procedures such as bleeding or perforation but also to decrease the associated costs of the pathological examination. To date, various image-enhanced endoscopy systems have been developed to improve polyps in clinical practice. Many papers have reported that AI can be used to help identify traditional adenomas [24] other than serrated colon polyps. However, 20–30% of colorectal cancers originate from serrated lesions [25]. As, Hirata et.al [26] report in their study, even experienced professionals found it difficult to accurately distinguish between SSAs and HPs using magnifying colonoscopy [26]. As deep-learning research progress, artificial intelligence (AI) is widely used in the interpretation of medical images. Therefore, we conducted this study to show how to combine a data argumentation heuristic and existing deep-learning model AI can help healthcare institute to build an in-house differential diagnosis of serrated colon polyps with white-light images.

SSA is not as commonly observed as TA or HP; thus, the size of the dataset is limited. To address this issue, we propose a method that combines image preprocessing, TensorFlow, and Inception V4 to build a polyp classification model. Using our proposed method, we built a highly accurate classification model (avg. AUC = 97.87%), with a sample consisting of 257 images of hyperplastic polyps (HP), 423 images of SSA, and 60 images of TA under white light. It is worth noting that our method allows the endoscopist to build/use the model with a limited sample size using white light images from ordinary endoscopy, not NBI or magnified images. Our AI model with only white-light images outperform the SSA and TA classification model build in Nemoto et al. [10], which obtain accuracy ranges from 77 to 87%. This might be due to the fact we combine the data argumentation with deep-learning model. The data argumentation allows the deep-learning model to extract more features, thus, create a more accuracy AI model.

In contrast, one of the significant benefits of accurately classifying the polyp type is that it helps relieve the financial burden of patients. The cost of removing an SSA or TA is substantial, and surgery may lead to unwanted side effects, such as internal bleeding. Siau et al. [27] found that it takes approximately 3.1 years and 265 procedures for an endoscopist to be fully aware of the characteristics of the different lesions. Hence, our model can assist endoscopists in reducing the healthcare industry in their endoscopist training and execution times.

According to current literature, most AI models require the use of magnifying colonoscopy or the combination with NBI for optimal performance. In contrast, our model can achieve good results with simple white-light colonoscopy alone. This outcome limits the convenience and widespread applicability of their models [9]. One of the contributions of our study is that our AI model works better for white light than NBI images. This contradicts the results of Lui et al. [8] ’s meta-analysis. In their paper, the NBI images were superior to white light images (98% vs. 84% accuracy). We argue that our AI model is superior to those of previous studies in two ways: (1) unequal sampling and (2) image preprocessing. We first adopt unequal sampling to allow the AI model to extract features for the SSA. Then, in *Algorithm 1*, we enhance all of the features with image per processing. These two steps help the AI model extract the most features and build a more accurate classification model.

While our AI model exhibits a high accuracy rate with white light images, this study is not without limitations. Our first limitation is that all our data are collected in the Lin-Kou Chang Gung Memorial Hospital. This might limit our model generality in terms of race. Second, our AI model is trained with unequal sampling data sets, this might increase the difficulty of future updates of the model. Third, our sample is small, but it also is our study’s strong point. However, applying our method to an enlarged dataset is a possible future research direction. We also encourage future researchers to develop an experimental design that applies the k-fold method to validate the proposed AI model.

## Conclusion

### Conclusion and contribution of the study

In this study we proposed a AI modeling framework combining a data argumentation heuristic and a deep-learning model that can build an efficient AI model with small data set of 257 white-light images of HP, 423 white-light images of SSA, and 60 white-light images of TA. With the result of our white-light images data set, we can conclude that our model can effectively help physicians distinguish between TA, SSA, and hyperplastic polyps. Our deep learning model provided high sensitivity (avg. 98.62%), specificity (97.12%), and discriminability (avg. AUC = 97.87%). Our method also requires only open-source packages such as TensorFlow and programming languages such as Python. From the healthcare institution’s perspective, the proposed AI modeling framework requires only open-source packages such as TensorFlow with Inception V4 and programming languages such as Python. Furthermore, our procedure can construct an efficient model with a small data set of white-light images. The simplicity of the AI modeling method and input image requirement allows the healthcare industry to quickly implement our method to build its models or modify our method to meet its needs.

From the endoscopist perspective, since our model only requires highlight images for the junior endoscopist, it is more user-friendly than the model that requires NBI images. Furthermore, the in-house model can assist the healthcare industry in more efficiently training junior endoscopists to classify colonic polyps and reduce training time and cost correctly. For endoscopists, the deep learning model reduces their time and workload while executing coloscopy so that they can provide high-quality services to the patient.

### Limitation and future study

While our study can significantly help healthcare institution and endoscopist, this study is not without limitations. First, our model is based on images which needed to be collected by the physician. If the healthcare institution implement both Node-RED to automatically upload the coloscopy images during the examination section, and link the pathological report with the upload images, our AI model can automatically train and improved. Furthermore, our current AI model is based only on the images, researchers can also find another data argumentation method which can be applied to video and build a model that can classified and detect the polyps during the colonoscopy without human interruption. Future researchers can also develop a more powerful deep-learning algorithm which can execute the real-time polyp location and classification process during the endoscopy section.

## Data Availability

Based on the institutional review board of the Chang Gung Memorial Hospital, the data used in this study should only be analyzed by the authors named in this manuscript, and no other entity has access to the raw data. Hence, authors are not authorized to share data with other research communities and/or the general public. Readers who would like to obtain the data used in this study, may contact Dr. Chen at itochenyu@gmail.com or q122583@cgmh.org.tw.

## Declarations

### Use humantissue sample

This experiment uses only to de-identification retrospective images and did not involve human tissue or fluid samples. The informed consent is waived by the Institutional Review Board of the Chang Gung Memorial Hospital due to this study only uses de-identification retrospective images.

## References

[1] Gupta S, Lieberman D, Anderson JC, Burke CA, Dominitz JA, Kaltenbach T, Robertson DJ, Shaukat A, Syngal S, Rex DK (2020). Recommendations for follow-up after colonoscopy and polypectomy: a consensus update by the US Multi-society Task Force on Colorectal Cancer. Gastrointest Endosc.

[2] Sano W, Hirata D, Teramoto A, Iwatate M, Hattori S, Fujita M, Sano Y (2020). Serrated polyps of the colon and rectum: remove or not?. World J Gastroenterol.

[3] East JE, Atkin WS, Bateman AC, Clark SK, Dolwani S, Ket SN, Leedham SJ, Phull PS, Rutter MD, Shepherd NA. British Society of Gastroenterology position statement on serrated polyps in the colon and rectum. Gut 2017:gutjnl–2017. 10.1136/gutjnl-2017-314005 PMC5530473 28450390

[4] Brown JRG, Mansour NM, Wang P, Chuchuca MA, Minchenberg SB, Chandnani M, Liu L, Gross SA, Sengupta N, Berzin TM (2022). Deep learning computer-aided polyp detection reduces adenoma miss rate: a United States multi-center randomized tandem colonoscopy study (CADeT-CS trial). Clin Gastroenterol Hepatol.

[5] Lee J, Bae JH, Chung SJ, Kang HY, Kang SJ, Kwak MS, Seo JY, Song JH, Yang SY, Yang JI (2022). Impact of comprehensive optical diagnosis training using Workgroup serrAted polypS and polyposis classification on detection of adenoma and sessile serrated lesion. Dig Endoscopy.

[6] Hassan C, Spadaccini M, Iannone A, Maselli R, Jovani M, Chandrasekar VT, Antonelli G, Yu H, Areia M, Dinis-Ribeiro M (2021). Performance of artificial intelligence in colonoscopy for adenoma and polyp detection: a systematic review and meta-analysis. Gastrointest Endosc.

[7] Hirasawa T, Aoyama K, Tanimoto T, Ishihara S, Shichijo S, Ozawa T, Ohnishi T, Fujishiro M, Matsuo K, Fujisaki J (2018). Application of artificial intelligence using a convolutional neural network for detecting gastric cancer in endoscopic images. Gastric Cancer.

[8] Azam MA, Sampieri C, Ioppi A, Africano S, Vallin A, Mocellin D, Fragale M, Guastini L, Moccia S, Piazza C (2022). Deep learning applied to white light and narrow band imaging videolaryngoscopy: toward real-time laryngeal cancer detection. Laryngoscope.

[9] Teramoto A, Hamada S, Ogino B, Yasuda I, Sano Y (2023). Updates in narrow-band imaging for colorectal polyps: narrow-band imaging generations, detection, diagnosis, and artificial intelligence. Dig Endosc.

[10] Nemoto D, Guo Z, Peng B, Zhang R, Nakajima Y, Hayashi Y, Yamashina T, Aizawa M, Utano K, Lefor AK (2022). Computer-aided diagnosis of serrated colorectal lesions using non-magnified white-light endoscopic images. Int J Colorectal Dis.

[11] Canziani A, Paszke A, Culurciello E. An analysis of deep neural network models for practical applications. arXiv Preprint arXiv:160507678 2016.

[12] Jha D, Smedsrud PH, Johansen D, de Lange T, Johansen HD, Halvorsen P, Riegler MA (2021). A comprehensive study on colorectal polyp segmentation with ResUNet++, conditional random field and test-time augmentation. IEEE J Biomedical Health Inf.

[13] Tomar NK, Jha D, Bagci U, Ali S. TGANet: Text-guided attention for improved polyp segmentation. In: *International Conference on Medical Image Computing and Computer-Assisted Intervention: 2022*: Springer; 2022: 151–160. 10.1007/978-3-031-16437-8_15 PMC9912908 36780239

[14] Lin Y, Han X, Chen K, Zhang W, Liu Q (2024). CSwinDoubleU-Net: a double U-shaped network combined with convolution and swin transformer for colorectal polyp segmentation. Biomed Signal Process Control.

[15] Dong B, Wang W, Fan D-P, Li J, Fu H, Shao L. Polyp-pvt: polyp segmentation with pyramid vision transformers. arXiv Preprint arXiv:210806932 2021.

[16] Fan D-P, Ji G-P, Zhou T, Chen G, Fu H, Shen J, Shao L. Pranet: Parallel reverse attention network for polyp segmentation. In: *International conference on medical image computing and computer-assisted intervention: 2020*: Springer; 2020: 263–273.

[17] Hossain MB, Iqbal S, Islam MM, Akhtar MN, Sarker IH (2022). Transfer learning with fine-tuned deep CNN ResNet50 model for classifying COVID-19 from chest X-ray images. Inf Med Unlocked.

[18] Szegedy C, Ioffe S, Vanhoucke V, Alemi A. Thirty-first AAAI conference on artificial intelligence. *Association for the Advancement of Artifcial Intelligence, USA* 2017:1–3.

[19] McNeely-White D, Beveridge JR, Draper BA (2020). Inception and ResNet features are (almost) equivalent. Cogn Syst Res.

[20] Howard AG, Zhu M, Chen B, Kalenichenko D, Wang W, Weyand T, Andreetto M, Adam H. Mobilenets: efficient convolutional neural networks for mobile vision applications. arXiv Preprint arXiv:170404861 2017.

[21] Ren X, Lin W, Yang X, Yu X, Gao H. Data augmentation in defect detection of Sanitary ceramics in Small and Non-i.i.d datasets. IEEE Trans Neural Netw Learn Syst 2022, Pp. 10.1109/TNNLS.2022.3152245 35263260

[22] Zhang A, Lipton ZC, Li M, Smola AJ. Dive into deep learning. arXiv 2021. *arXiv preprint arXiv:210611342* 2021.

[23] Morson B. The polyp-cancer sequence in the large bowel. In.: SAGE Publications; 1974. 10.1177/00359157740676P115 PMC1645739 4853754

[24] Pannala R, Krishnan K, Melson J, Parsi MA, Schulman AR, Sullivan S, Trikudanathan G, Trindade AJ, Watson RR, Maple JT (2020). Artificial intelligence in gastrointestinal endoscopy. VideoGIE.

[25] Rosty C, Hewett DG, Brown IS, Leggett BA, Whitehall VL (2013). Serrated polyps of the large intestine: current understanding of diagnosis, pathogenesis, and clinical management. J Gastroenterol.

[26] Hirata D, Kashida H, Matsumoto T, Ebisutani C, Teramoto A, Iwatate M, Hattori S, Fujita M, Sano W, Komeda Y (2023). A Multicenter prospective validation study on selective endoscopic resection of Sessile Serrated lesions using magnifying Colonoscopy in Clinical Practice. Digestion.

[27] Siau K, Hodson J, Valori RM, Ward ST, Dunckley P (2019). Performance indicators in colonoscopy after certification for independent practice: outcomes and predictors of competence. Gastrointest Endosc.

